# Impact of lockdown on Covid-19 case fatality rate and viral mutations spread in 7 countries in Europe and North America

**DOI:** 10.1186/s12967-020-02501-x

**Published:** 2020-09-02

**Authors:** Maria Pachetti, Bruna Marini, Fabiola Giudici, Francesca Benedetti, Silvia Angeletti, Massimo Ciccozzi, Claudio Masciovecchio, Rudy Ippodrino, Davide Zella

**Affiliations:** 1 Elettra Sincrotrone Trieste-Area Science Park, Trieste, Italy; 2 Department of Physics, University of Trieste, Via Valerio 2, Trieste, Italy; 3 Ulisse BioMed-Area Science Park, Trieste, Italy; 4 Department of Medicine, Surgery and Health Science, University of Trieste, Trieste, Italy; 5 Institute of Human Virology, Department of Biochemistry and Molecular Biology, School of Medicine, University of Maryland, Baltimore, USA; 6 Medical Statistic and Molecular Epidemiology Unit, University of Biomedical Campus, Rome, Italy; 7 Member of the Global Virus Network, Baltimore, USA

**Keywords:** SARS-CoV-2, Case fatality rate, Mutation, Europe, US, COVID-19, Lockdown strategy, Testing capacity

## Abstract

**Background:**

Severe acute respiratory syndrome CoV-2 (SARS-CoV-2) caused the first coronavirus disease 2019 (COVID-19) outbreak in China and has become a public health emergency of international concern. SARS-CoV-2 outbreak has been declared a pandemic by WHO on March 11th, 2020 and the same month several Countries put in place different lockdown restrictions and testing strategies in order to contain the spread of the virus.

**Methods:**

The calculation of the Case Fatality Rate of SARS-CoV-2 in the Countries selected was made by using the data available at https://github.com/owid/covi-19-data/tree/master/public/data. Case fatality rate was calculated as the ratio between the death cases due to COVID-19, over the total number of SARS-CoV-2 reported cases 14 days before. Standard Case Fatality Rate values were normalized by the Country-specific ρ factor, i.e. the number of PCR tests/1 million inhabitants over the number of reported cases/1 million inhabitants. Case-fatality rates between Countries were compared using proportion test. Post-hoc analysis in the case of more than two groups was performed using pairwise comparison of proportions and *p* value was adjusted using Holm method. We also analyzed 487 genomic sequences from the GISAID database derived from patients infected by SARS-CoV-2 from January 2020 to April 2020 in Italy, Spain, Germany, France, Sweden, UK and USA. SARS-CoV-2 reference genome was obtained from the GenBank database (NC_045512.2). Genomes alignment was performed using Muscle and Jalview software. We, then, calculated the Case Fatality Rate of SARS-CoV-2 in the Countries selected.

**Results:**

In this study we analyse how different lockdown strategies and PCR testing capability adopted by Italy, France, Germany, Spain, Sweden, UK and USA have influenced the Case Fatality Rate and the viral mutations spread. We calculated case fatality rates by dividing the death number of a specific day by the number of patients with confirmed COVID-19 infection observed 14 days before and normalized by a ρ factor which takes into account the diagnostic PCR testing capability of each Country and the number of positive cases detected. We notice the stabilization of a clear pattern of mutations at sites nt241, nt3037, nt14408 and nt23403. A novel nonsynonymous SARS-CoV-2 mutation in the spike protein (nt24368) has been found in genomes sequenced in Sweden, which enacted a soft lockdown strategy.

**Conclusions:**

Strict lockdown strategies together with a wide diagnostic PCR testing of the population were correlated with a relevant decline of the case fatality rate in different Countries. The emergence of specific patterns of mutations concomitant with the decline in case fatality rate needs further confirmation and their biological significance remains unclear.

## Background

SARS-CoV-2, the etiologic agent of the current global pandemic, is an enveloped positive-sense single-stranded RNA (+ssRNA) virus, that belongs to the *Betacoronavirus* genus and to the Coronaviridae family, which is broadly distributed in humans and other mammals [1–3]. Also, during the last decades, other newly emerged coronaviruses have caused respiratory infections with pandemic potential, such Severe Acute Respiratory Syndrome coronavirus (SARS-CoV) and the Middle East Respiratory Syndrome coronavirus (MERS-CoV).

Similarities of clinical features between previous *Betacoronavirus* infections and SARS-CoV-2 have been noted. Moreover, full genome sequencing has shown that it is closely related to SARS-CoV, both viruses have about 80% similarity and their genomes consist of six major open-reading frames (ORFs) plus a number of other accessory genes. Also, molecular modelling indicated similarities between their receptor-binding domains. The spike protein, that presents the most immunogenic determinants of the virus, has been shown to bind the same SARS-CoV receptor (the angiotensin converting enzyme 2 receptor, ACE2) in order to invade cells, suggesting a similar pathogenic mechanism.

As of April 30th, 2020 there were approximately 3.1 M confirmed cases of COVID-19 worldwide and more than 217.000 infection-related deaths. SARS-CoV and MERS-CoV have caused more than 10,000 cumulative cases in the past two decades, with mortality rates of 9,6% for SARS-CoV and 37% for MERS-CoV, respectively [4–7].

Although SARS-CoV-2 is less lethal than MERS-CoV, as many as 20% of the infected people develop rapidly a severe disease characterized by interstitial pneumonia and acute respiratory distress syndrome that can ultimately lead to death. This is particularly reported in elderly and in people with underlying medical conditions. However, most of the patients remain asymptomatic or develop mild symptoms, like fever and dry cough, followed then by breathing difficulties (dyspnea), and bilateral ground-glass opacities on chest CT scans, indicating that the target cells are located in the lower airways [8].

Nowadays, the main goal is to identify an effective treatment and a vaccine against SARS-CoV-2 and to found effective diagnostics, sociological and public health strategies to reduce the spread of the virus, ensuring a faster economic recovery.

This study aims to compare the effectiveness of the different lockdown strategies, the need of a considerable diagnostic PCR testing capability as well as the impact of the representative viral strains isolated in each Country presented here. To this purpose, we focused our study on Italy, Spain, France, Germany, UK, Sweden and United States, broadening our previous analysis of SARS-CoV-2 variants [9]. The characterization of SARS-CoV-2 variants might also significantly contribute to the design of effective therapies, vaccines and novel diagnostics tools.

## Methods

Case fatality rate (CFR) represents the proportion of cases who eventually die from a disease over the diagnosed cases of disease (https://ourworldindata.org/mortality-risk-covid). Once an epidemic has ended, CFR is calculated as (deaths cases/infected cases). However, while an epidemic is still ongoing, as it is the case with the current novel coronavirus outbreak, this formula does not represent the true case fatality rate and might be off by orders of magnitude. Diagnosis of viral infection will precede recovery or deaths by days to weeks and the number of death should therefore be compared to the past case counts—accounting for this delay increasing the estimate of the case fatality rate [10].

To calculate CFR, we used the following formula:

$$ {\text{CFR }} = {\text{ deaths at day x }}/{\text{ cases at day x}} - \left\{ {\text{T}} \right\} $$

where T: average time period from case confirmation to death.

Therefore, in our study, CFR was calculated as the ratio between the death cases due to COVID-19, over the total number of SARS-CoV-2 reported cases 14 days before, as previously described [11]. We normalized these rates among different Countries, considering the different policies in terms of number of testing/million inhabitants, and at the same time considering the different incidence of the infection taking into account the number of cases/million inhabitants. A corrective Country-specific ρ factor was defined as the ratio between the number of PCR tests/1 million inhabitants and the number of reported cases/1 million inhabitants (data obtained from https://www.worldometers.info/coronavirus/#countries). Standard CFR values were normalized by the Country-specific ρ factor. CFR between Countries were compared using proportion test. Post-hoc analysis in the case of more than two groups was performed using pairwise comparison of proportions and p-value was adjusted using Holm method.

We also analyzed 487 full-length genomic sequences of SARS-CoV-2 from GISAID database. Sequenced specimens were collected from December 2019 to April 2020, from the following Countries: Germany, Italy, Spain, France, UK, Sweden and USA. NC_045512.2 genome deposited in the GenBank has been used as SARS-CoV-2 reference genome. Muscle and Jalview software were used for genomes alignment and analysis.

## Results and discussions

### CFR comparison in different Countries

**Fig. 1:**
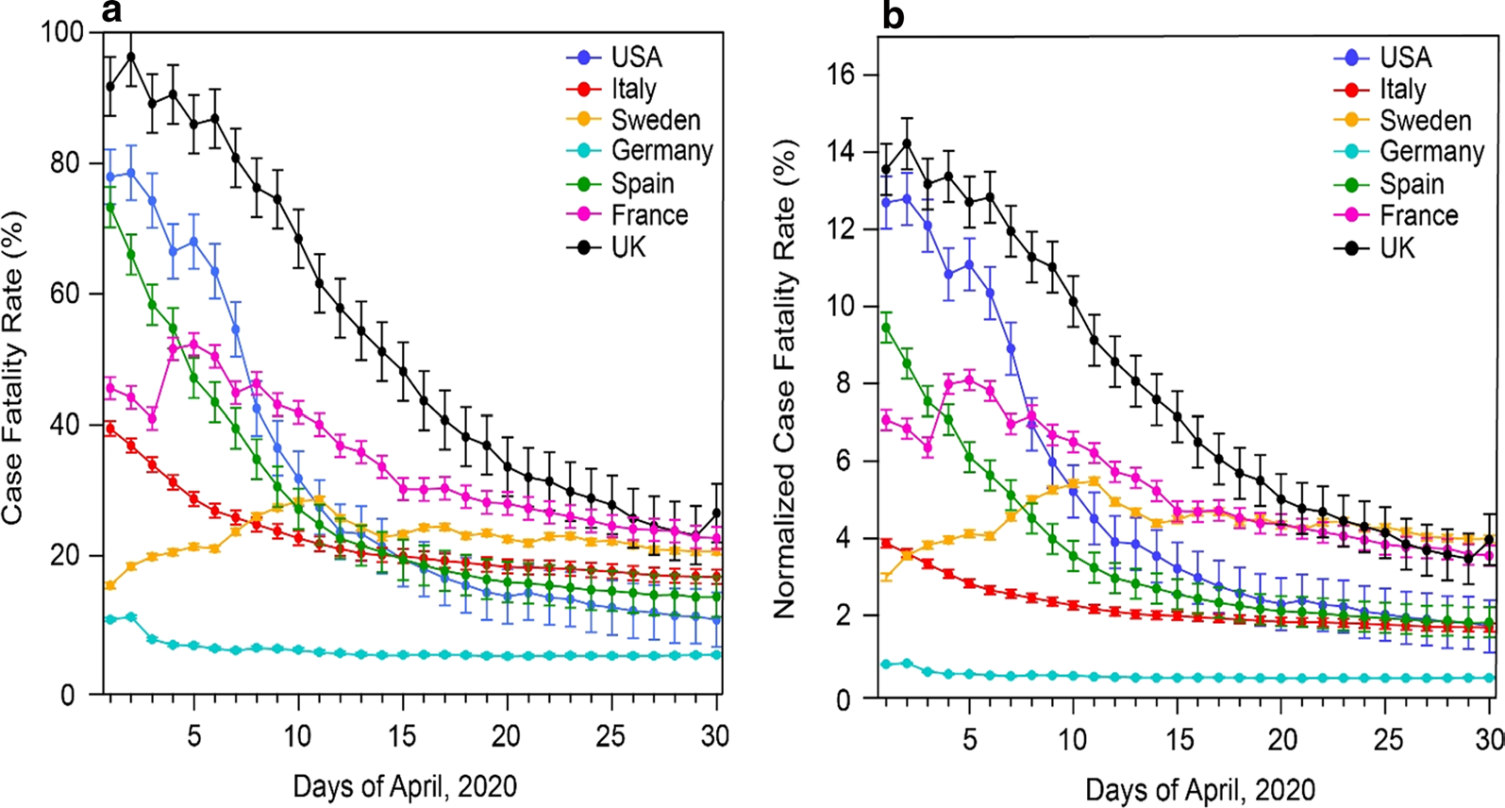
**a** Calculated case fatality rate curves for USA, Italy, Sweden, Germany, Spain, France and UK as explained by Baud and colleagues [11]. Bars indicate the 95% of confidence interval. **b** Case fatality rate of **a** normalized by the ρ factor, i.e. by the number of PCR tests performed per 1 M population over positive cases per 1 M population up to the 30th of April, 2020. Bars indicate the 95% of confidence interval. The normalization leads to the formation of three main groups: group 1 includes Germany, group 2 includes Italy, USA and Spain and group 3 includes UK, France and Sweden

**Fig. 2:**
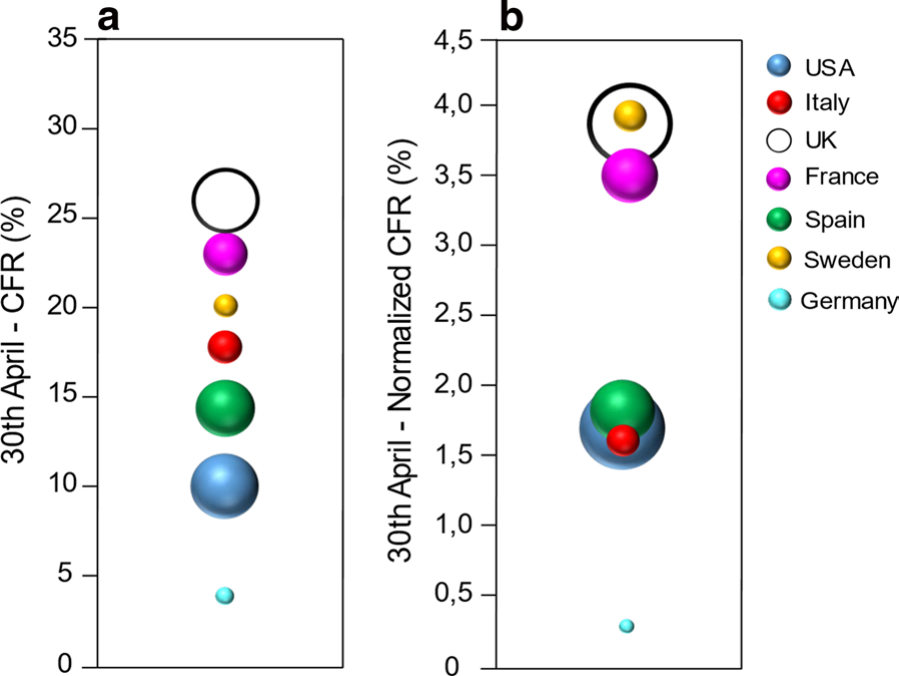
Bubble plot representation of the 30th of April Case Fatality Rate (**a**) and of the 30th of April Normalized Case Fatality Rate of USA (in light blue), Italy (in red), of UK (black line), France (in magenta), Spain (in green), Sweden (in yellow) and Germany (in cyan). In **a**, the CFRs are distributed within a large range of values, whereas in **b** the normalized CFRs values are clustered in three well-distinct groups: Germany forms the first group, Italy, Spain and USA the second group and, finally, Sweden, UK and France the third group with the higher normalized CFR value

Mortality calculations during the epidemics are difficult, mostly due to calculation biases: during the initial period of the epidemic, many patients were diagnosed with COVID-19 only after developing critical illness or even at the time of death, whereas asymptomatic or paucisymptomatic patients were untested, leading to an underestimation of the denominator [11]. Additional significant biases affect mortality curves: to name a few, the parameters used for death counting, the rigidity of lockdown measures, population age. Over time Countries started adopting better policies for diagnostic PCR testing and lockdown strategies, and consequently the spread of the virus was better monitored and the data were more carefully determined. We chose to analyze the Country-specific data relative to the number of COVID-19 deaths in April 2020, when some of the initial biases were likely attenuated, using the method described [11]. The number of deaths of a specific day was divided by the total number of infected cases reported 14 days before. This method considers the fact that 14 days are the average lag time estimated between the first symptoms to death [12]. The data analyzed for Italy, France, Germany, Spain, UK, Sweden and USA are reported in Fig. 1a. For all Countries we observed a decrease in the CFR values over time, with the exception of Germany (that maintains a very low value overall) and Sweden (where no decrease is observed). We identified two critical elements that might affect CFR among these Countries: (a) the number of PCR tests made and (b) the total number of positive cases for each Country. Since the second parameter (b) depends on the first parameter (a), we introduced a corrective Country-specific factor ρ = a/b, that was later used to normalize the CFR previously calculated (Table 1). Data obtained through this normalization model are reported in Fig. 1b. By taking only the data calculated on the 30^th^ of April and representing them in a bubble plot (Fig. 2), we clearly identify the presence of three clusters of Countries. Group 1 includes Germany and has a very low normalized CFR (0.31% CI (95%) [0.29 : 0.33] on April 30th 2020). Group 2 includes Italy, USA and Spain and has an intermediate value of normalized CFR (1.62% CI (95%) [1.51:1.72]; 1.65% CI (95%) [0.97:2.33]; 1.76% CI (95%) [1.36:2.15], respectively, on April 30th 2020). Group 3 includes France, Sweden and UK (3.49% CI (95%) [3.23:3.76]; 3.92% CI (95%) [3.83:4.02]; 3.90% CI (95%) [3.25:4.27], on April 30th 2020). The difference among cluster’s CFR (respectively 0.31% vs 1.68% vs 3.78%) was statistically significant (p < 0.001). Also, all pairwise comparisons were significant (p-adjusted with Holm method < 0.001).

**Table 1:** Country-specific data showing number of PCR tests and cases per million inhabitants and corrective factor ρ = a/b

	Italy	Spain	France	Germany	UK	Sweden	USA
PCR Tests/1 M inhabitans (a)	36244	41332	16856	30400	19026	11833	22545
Cases/1 M inhabitans (b)	3505	5311	2596	1984	2807	2250	3665
Corrective factor (ρ)	10.34	7.78	6.49	15.32	6.78	5.26	6.15

This result could be furtherly refined by considering the variability of the lag time due to patients age, i.e. older people (> 70 y.o.) have a lower lag time [12] compared to others. However, even if the daily number of death patients divided per age is available for each Country, we could not provide in this study a further normalization of the CFR taking into account patients age, since a similar daily database of infected people divided per age is not publicly available. Anyway, since the infection mostly leads to death older people or those that have ongoing severe illnesses (i.e. cardiovascular diseases, diabetes, cancer), we can speculate that the overall estimation of the CFR is driven by this class of patients. Therefore, the observed CFR curves observed among different Countries through the introduction of an innovative corrective factor ρ, might be explained mainly by the different policies that were enacted by each Country. To further support this hypothesis, we note that in Countries of group 3 where lockdown was not put in place (i.e. Sweden) or it was adopted late, and less SARS-CoV-2 PCR tests were executed (i.e. in UK and France), normalized CFR is higher than in the other groups. Although further data are needed to refine the CFR estimation, we improved the CFR estimate by using a new corrective factor which considers two important variables (number of positives and number of PCR tests performed). In fact, several sources of variability affect CFR but for modifiable confounding factors, a standardization process could help to reduce the biases, improving the interpretability and comparability of CFR across Countries.

### Lockdown impact on viral mutation spread

A database of 487 genome sequences isolated from patients infected with SARS-CoV-2 in Italy, Spain, Germany, France, UK, Sweden and USA has been randomly collected from the GISAID database, aligned and compared to the SARS-CoV-2 reference genome. A total of 27 genomes were considered in January 2020, 91 in February 2020, 210 in March 2020 and, finally, 159 genomes in April 2020. We analyzed 54 genome samples collected in Italy, 61 in Spain, 62 in Germany, 52 in France, 80 in UK, 50 in Sweden and 128 in the United States (Table 2).

**Table 2:** Sequenced genomes selection for different geographic areas and time of collection

	Italy	Spain	France	Germany	UK	Sweden	USA	Tot
January 2020	3	0	8	1	2	0	13	27
February 2020	5	7	11	12	25	2	29	91
March 2020	36	35	24	23	27	25	40	210
April 2020	10	19	9	26	26	23	46	159
Tot	54	61	52	62	80	50	128	487

**Fig. 3:**
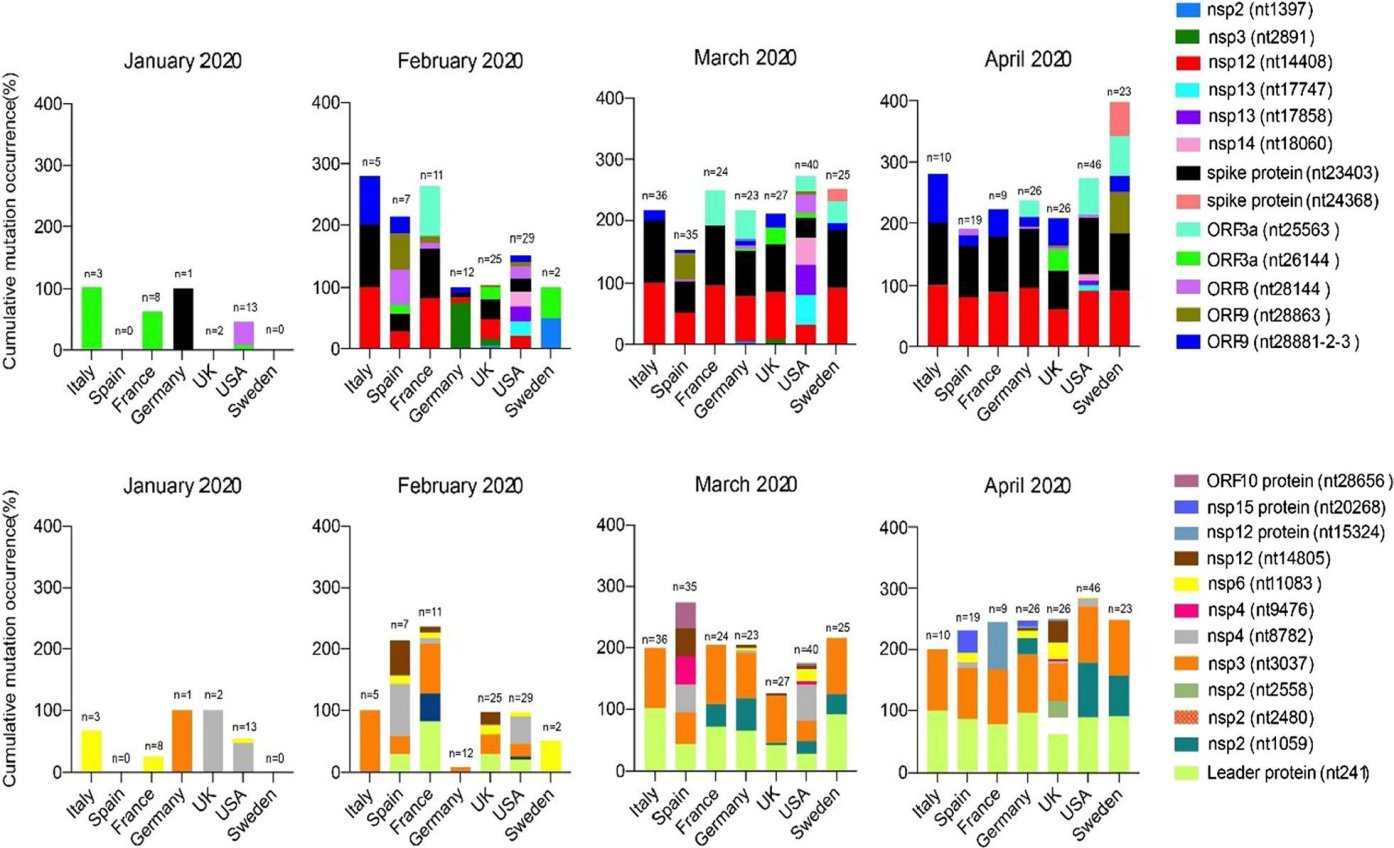
SARS-CoV-2 recurrent mutations occurrence over time, divided per geographic area. The graph reports evolution of nonsynonymous (top) and silent (bottom) mutation patterns from January 2020 to April 2020 in Italy, Spain, France, Germany, UK, Sweden and in the USA. The frequency of each mutation in each country and for each month was normalized to 100%, i.e. to the total number of genomes analyzed in that frameshift and collected in that specific country. Recurrent nonsynonymous mutation pattern is characterized by the occurrence of mutations at nt14408, nt 23403 and nt28881–28882–28883 (RdRp and spike protein, respectively), while the most found silent mutations are at nt241 and nt3037 (localized in the leader protein and in the nsp3)

We studied the evolution of the mutation patterns in the selected Countries from January to April 2020, and we reported only the recurrent mutations occurring more than 10 times in the time range considered, as described elsewhere [9]. The occurrence of each mutation in a specific Country has been normalized by the number of genomes collected in that geographic area for each timeframe, dividing the silent by the non-silent mutations (Fig. 3). Interestingly, the number of nonsynonymous mutations increases over time during the spread out of Asia, and appears to stabilize in April (Fig. 3, top panel). The pattern of nonsynonymous mutations changes quite dramatically from January to February, when such mutations appeared for the first time. More in detail, part of the genomes analyzed in January 2020 belong to patients infected in China or to patients in close contact to those travelling or coming back from Asia. In February, most Countries decided to suspend flights at first from and to China and, after, only few communications were maintained between nations and during that month locally transmitted outbreak cases occurred. We observed a pattern of recurrent mutations which reached a homogeneous distribution across the different Countries in March 2020. This observation is confirmed also in April 2020 in all the analyzed Countries. It is likely that lockdown policies implemented in this period greatly reduced further viral spread from Asia and hampered mixing of SARS-CoV-2 strains among Countries. We observed a similar pattern for silent mutations (Fig. 3, bottom panel).

Overall, our data show a number of silent mutations (nt241, nt3037) and nonsynonymous mutations (nt14408, nt23403 and nt28881–28882–28883) (Fig. 3). Among the nonsynonymous mutations, we note the occurrence of an already observed mutation at position 14408, which is located in the viral RNA-dependent RNA-polymerase (RdRp gene), a key component of the replication/transcription machinery [9]. This mutation (Fig. 3, depicted in red) emerged in February 2020 and is quite homogeneously distributed across all the Countries analyzed. This is also observed for a mutation occurring in the spike protein (nt23403, Fig. 3, depicted in black) and to a minor extent for a mutation in the nucleocapsid phosphoprotein (nt28881–28882–28883, Fig. 3, depicted in blue). The occurrence of the mutation in the RdRp (nt14408) is always associated with that of the spike protein (nt23403), of the nsp3 mutation (nt3037) and of the mutation in the leader protein (nt241). A different pattern of hotspot mutations characterized viral genomes detected in patients from the United States. In February we initially detected three novel mutations (in position 17747, 17858 and 18060), that were not found elsewhere. These mutations were found predominantly in the viral genomes sequenced in Washington State (USA). The occurrence of this isolated pattern over time reflects the viral spreading of a more “European-like” strain (nt241, nt3037, nt14408 and nt23403) in the rest of the US. Overall, the occurrence of this “European-like” group varies from 32.5% of analyzed genomes (in USA) to 100% (in Italy). Our data confirm the previous observations made by Korber et al. [13], when the authors hypothesized that this mutation group, associated with the G clade, could enhance viral fitness, possibly due to the nt23403 mutation that triggers a significant amino acid substitution in a strongly immunogenic linear epitope of Spike protein, which might affect neutralizing antibodies sensitivity.

### Emerging of new mutations

We noted the emergence of other recurrent mutation sites over time, both nonsynonymous (nt25563, nt28863) and silent (nt2480, nt2558, nt9476, nt15324, nt20268 and nt28656). The nonsynonymous mutations occur in the ORF3a and ORF9 (nucleocapsid phosphoprotein), causing the amino acid mutation Q56H (glutamine to histidine) and S197L (serine to leucine). All these mutations are found in most Countries and they are not exclusively reported in a specific geographic area. An additional recurrent mutation has been detected exclusively in genomes from Swedish at nt24368 (G to T transition); this mutation, which is located in the spike protein sequence, appeared in March (carried by 20% of genomes analyzed) and its frequency more than doubled in April (52% of genomes analyzed). This mutation triggers an amino acid substitution at position 936, from an aspartic acid to a tyrosine, with a significant shift in terms of isoelectric point from 2.85 to 5.64. D936 residue in SARS-CoV-2 Spike protein corresponds to the E918 residue of the homologue protein of SARS-CoV, and it is located in the heptad repeat 1 (HR1) domain [14, 15]. Heptat repeat 1 interacts with heptad repeat 2 (HR2) domain and form a six-helix bundle fusion core, able to bring viral and cellular membranes in close proximity, promoting fusion and infection of host cell [16, 17]. This makes HR1 and HR2 good target candidates for drug design. Recently, D936 (site of the recurrent mutation) has been proved to bind to R1185 of the heptad repeat 2 (HR2) domain through a salt bridge. Additional studies are required to further characterize if G936 mutant, present in April in more than half of Swedish genomes analyzed, could provide some beneficial advantages in terms of viral fitness, as observed for mutation nt23403 [13]. Among the Countries in the different groups there are no significant differences in the distribution of mutations, since the recurrent mutation pattern is comparable among different Countries (Fig. 3, top panel). The only significant difference is the newly emerged mutation nt24368, that in our database was detected only in the genomes analyzed in Sweden.

## Conclusions

By normalizing the CFR by the ρ factor, we divided the analyzed Countries in three groups with an increased estimated CFR: group 1 is represented by Germany, group 2 by Italy, Spain and USA and group 3 by Sweden, France and UK. Groups 1 and 2 include Countries that adopted strict lockdown strategies and/or have a wide testing capability, whereas group 3 is formed by Countries that have adopted lockdown restrictions later (or have not at all) and/or did not perform an extensive diagnostic PCR testing. A decreasing trend of case fatality rate has been observed among most Countries. There are several direct factors that might contribute to this decline, such as health service’s ability to cope with COVID-19 patients, increased and improved viral testing and tracing, efficacy of the different lockdown strategies, herd immunity development, influence of age on the affected population, variation in viral contagiousness and lethality. We observe that, after the rapid emergence and diffusion of recurrent mutations in February and March, a specific mutation pattern has stabilized by April 2020 in all the Countries analyzed. This pattern is comprised of mutations nt241, nt3037, nt14408 and nt23403. In Sweden we report the occurrence of a unique nonsynonymous mutation in the spike protein (nt24368) which has been found in more than 50% of genomes. The emergence of specific patterns of mutations concomitant with the decline in case fatality rate needs further confirmation and the biological significance of such mutations remains unclear.

## Data Availability

Data available in the GISAID database and in a public repository that does not issue datasets with DOIs.

## Abbreviations

CFR: case fatality rate
UK: United Kingdom
WHO: World Health Organization
